# Exploring the Relationship between Self-Rated Health and Unmet Cancer Needs among Sexual and Gender Minority Adolescents and Young Adults with Cancer

**DOI:** 10.3390/curroncol30100671

**Published:** 2023-10-20

**Authors:** Nina Francis-Levin, Lauren V. Ghazal, Jess Francis-Levin, Bradley Zebrack, Meiyan Chen, Anao Zhang

**Affiliations:** 1 Division of Endocrinology, Metabolism & Diabetes, Michigan Medicine, University of Michigan, Ann Arbor, MI 48109, USA; ninalev@umich.edu; 2 School of Nursing, University of Rochester, Rochester, NY 14642, USA; lauren_ghazal@urmc.rochester.edu; 3 Institute for Social Research, University of Michigan, Ann Arbor, MI 48104, USA; jessfran@umich.edu; 4 School of Social Work, University of Michigan, Ann Arbor, MI 48109, USA; zebrack@umich.edu (B.Z.); myanchen@umich.edu (M.C.)

**Keywords:** sexual and gender minority, adolescent and young adult cancer, self-rated health

## Abstract

This study evaluates the unmet needs of sexual and gender minority (SGM) adolescent and young adult (AYA) cancer survivors by comparing SGM AYA self-rated health (SRH) scores to their non-SGM (i.e., cisgender/heterosexual) counterparts. The Cancer Needs Questionnaire—Young People (CNQ-YP) and self-rated health measures were used to assess unmet needs in AYAs aged 15–39 who had been diagnosed with cancer in the previous ten years (*n* = 342). Participants were recruited from a National Cancer Institute (NCI) Comprehensive Cancer Center registry using the modified Dillman’s method. Self-reported sexual orientation and gender identity (SO/GI) data were collected. Independent *t*-tests were used to test between-group differences in unmet needs and Pearson’s chi-square test was used to determine the difference in SRH scores between SGM and non-SGM AYA cancer survivors. SGM AYA cancer survivors reported greater mean needs than their non-SGM counterparts across all six domains and reported significantly greater needs in the domains of Feelings and Relationships, *t*(314) = −2.111, *p* = 0.036, Information and Activities, *t*(314) = −2.594, *p* = 0.009, and Education, *t*(207) = −3.289, *p* < 0.001. SGM versus non-SGM SRH scores were significantly different, indicating that a higher percentage of SGM AYAs reported poor/fair health compared to those who were non-SGM. Unmet life and activities needs were negatively associated with AYA cancer survivors’ SRH, whereas unmet work needs were positively associated with AYA cancer survivors’ SRH. An AYA’s gender identity (SGM versus non-SGM) was not a moderator. SGM AYAs are an understudied group within an already vulnerable patient population. Unmet psychosocial needs related to one’s feelings and relationships, and information and activity needs merit further research to develop tailored interventions that reflect the experiences of SGM AYAs.

## 1. Introduction

Adolescent and young adult (AYA) cancer patients and survivors (15 to 39 years old) are a vulnerable population who face distinct age-related challenges throughout their disease trajectory [1,2]. Based on data from the National Cancer Institute (NCI), approximately 4.5% (over 89,500) of new cancer cases are among the AYA population in 2022, and nearly 633,000 of survivors in the United States are below the age of 39 [2,3,4]. On top of the many common challenges confronting cancer survivors across the age spectrum (e.g., treatment-related side- and late-effects), AYA cancer survivors face additional life-stage-driven concerns, including but not limited to occupational disruptions, financial burden, compromised infertility, intimate relationship delays and challenges, sexual dysfunction, and substance misuse, among others [5,6,7,8,9,10]. Accordingly, AYAs face unmet psychosocial needs in the domains of health and healthcare, informational needs, communication and relationships, sexual and reproductive health, emotional wellbeing and coping skills, vocational disruptions and financial burden [11,12,13].

Sexual and gender minorities (SGM)—i.e., those who identify as lesbian, gay, bisexual, transgender, nonbinary, queer, and/or other non-cis-heteronormative identities—are an underserved population of cancer survivors [14,15,16]. AYAs who sit at the intersection of multiple marginalized identities (i.e., AYA and SGM) may face compounded challenges [17]. Including the above-mentioned challenges for all AYAs, SGM AYAs also experience a higher risk for certain cancers, lower rates of prophylactic screening, fear of discrimination or denial of care by healthcare providers, internalized homophobia, increased substance use, and increased psychological distress [15,18,19,20,21,22,23]. Beyond the unmet needs facing cisgender/heterosexual AYAs, SGM AYAs face further unmet needs related to identity development, including disclosing SGM identities to providers, navigating stigmatization, and accessing safe and relevant care for sexual-, reproductive-, and gender-health before, during and after active treatment [24,25,26].

In 2021, about 20.8% of emerging adults, born 1997–2003, also referred to as “Gen Z”, and 10.5% of young adults, born 1981–1996, also known as “Millennials”, identified as SGM. These data represent a major increase in self-identification and identity disclosure when compared to the rate of US adults born before 1980 who self-identified with/disclosed SGM identities (7.6%) [27]. Therefore, it is reasonable to believe that SGM AYAs are likely to comprise a sizable fraction of the AYA survivor population, highlighting the epidemiological significance of attending to the SGM AYA population. The SGM population as a vulnerable group, compounded by the AYA age-range (another risk factor), have been consistently connected with a broad domain of compromised physical and behavioral health outcomes, including, but not limited to, psychological distress, psychosocial functioning, cancer-related quality of life, and general wellness [28,29,30]. One salient patient-reported outcome (PRO), however, that has not been comprehensively studied among SGM AYA cancer survivors is self-rated health (SRH) [31].

SRH is a patient-centered measure of an individual’s general health status, which integrates the biopsychosocial and functional aspects of their health, including cultural beliefs and health behaviors [32,33]. Despite the brevity of SRH as a single-question measure of global health, studies have documented the predictive power of SRH in relation to individuals’ morbidity and mortality rates across diverse populations [34,35]. Notably, specifically for the cancer population, SRH has been extensively validated across the sexes, age spectrum, racial/ethnic groups, and cancer stages, endorsing the broad psychometric applicability of SRH [36,37,38]. Several studies have utilized the SRH measure among the AYA cancer population, suggesting its validity for this population [39,40]. Yet, limited investigations into risk factors impacting AYA cancer survivors’ SRH exist, with insufficient examination specifically among SGM AYA.

As such, in this study, our goal was to use the multi-dimensional unmet needs measure for AYAs with cancer, the Cancer Needs Questionnaire—Young People (CNQ-YP) to evaluate the unmet needs of SGM AYA cancer survivors by comparing between-group SRH score differences to those of their non-SGM (i.e., cisgender/heterosexual) counterparts [41,42]. We hypothesized that SGM AYAs would have significantly higher unmet needs across all domains and lower SRH compared to their non-SGM counterparts.

## 2. Materials and Methods

### 2.1. Study Design

We used a cross-sectional survey design and evaluated the unmet psychosocial needs of AYAs living with a cancer diagnosis who are receiving or have received care at an NCI-Designated Comprehensive Cancer Center (Unversity of Michigan Rogel Cancer Center at Michigan Medicine). Specifically in this project, we had the following main objectives: (1) to describe the unmet psychosocial needs of AYA cancer survivors; (2) to evaluate the difference between SGM and non-SGM AYA cancer survivors’ SRH and unmet psychosocial needs; and (3) to preliminarily explore the unmet cancer care needs of AYAs with cancer in relation to their SRH, especially considering SGM status as a potential moderator. The study was approved by the University of Michigan IRBMED (HUM00180540).

### 2.2. Participant Recruitment and Study Procedure

**Figure 1:**
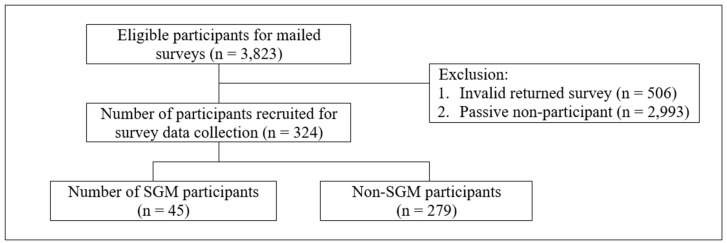
Flow chart for the recruitment of adolescent and young adult cancer survivors.

Figure 1 describes the recruitment process. For a participant to be considered eligible for the study, a respondent must have been between the ages of 15 and 39 years old, with a current diagnosis of cancer, or a survivor of cancer diagnosed within the previous 10 years, with at least one appointment for cancer care at the study institution. In accordance with the definition of the NCI, an individual is considered as a cancer survivor from the time of diagnosis [43]. Therefore, we included participants in both active treatment and post-treatment survivorship stages in the current project. Upon approval from the institutional medical IRB, our team used convenience sampling within the cancer registry at Michigan Medicine to identify potential participants. The registry query yielded medical record number (MRN), class of case (role of the institution in patient’s case), current age (date of birth), date of first and last contact with Michigan Medicine first and last name, primary cancer site, ICD-O-3 Histology and Behavior code, current address, and vital status. A total of *n* = 3823 potential participants were identified in this manner and were contacted via postal mail.

As a first step, we mailed surveys and consent forms (*n* = 3823) to participants between August 2021 and February 2022 using the modified Dillman’s method [44] Participants opted to complete the survey by paper or online via Qualtrics. As a result of the survey dissemination, we received a total of *n* = 830 returned mailers, including *n* = 506 invalid returns (e.g., address no longer active) and *n* = 324 valid returns (*n*= 318 by paper, *n* = 6 by Qualtrics). Participants self-reported sexual orientation and gender identity (SO/GI) using a ‘select all that apply’ format informed by methodological considerations for SO/GI self-reported data [45]. Participants who selected SO/GI items other than cisgender and/or heterosexual were categorized as SGM (*n* = 45).

As a final step, the first author (N.F.-L.) and a research assistant tracked and documented all returned surveys and extracted data to a database internally stored in a University of Michigan firewall-protected server. The study’s principal investigator (A.Z.) randomly selected and double-checked the data input of 25% of all valid surveys, revealing a 99.9% inter-extractor reliability rate. All enrolled study participants were tracked and reported in the clinical and translational oncology research platform—OnCore. Participants were mailed a $15 USD incentive to thank them for participating.

### 2.3. Measurement

#### 2.3.1. Demographic and Clinical Variables

Demographic and clinical variables were collected as potential covariates. We obtained participants’ age (in years), current cancer treatment status (1 = active treatment, 2 = within 1 year post-treatment, 3 = 1–3 years post-treatment, 4 = 3–5 years post-treatment, and 5 = 5 or more years post-treatment), and race/ethnicity (which was recoded into non-Hispanic White versus others given the distribution of this variable). SO/GI items included gender identity (1 = Women/Girl, 2 = Man/Boy, and 3 = Transfeminine, 4 = Transmasculine, 5 = Nonbinary, 6 = Two-spirit, 7 = Cisgender, 8 = Open-response/free-text, and 9 = Prefer not to say), and sexual orientation (1 = Lesbian, 2 = Gay, 3 = Bisexual, 4 = Pansexual, 5 = Straight/heterosexual, 6 = Queer, 7 = Open-response/free-text, 8 = prefer not to say). For race/ethnicity and SO/GI items, participants were instructed to select all that apply and are reported as such. Individuals who selected “Prefer not to say” were included as SGM.

#### 2.3.2. The Unmet Needs of AYAs with Cancer

The unmet cancer care needs of the participants were measured using the Cancer Needs Questionnaire—Young People (CNQ-YP). The CNQ-YP was developed to evaluate the unmet psychosocial and supportive care needs of AYA cancer survivors using a comprehensive process of literature review, focus groups with AYAs, and feedback from health care providers, researchers, and other professionals [41,42]. The CNQ-YP contains a total of 112 questions and covers 6 main areas of (unmet) needs: (1) Treatment Environment and Care, (2) Feelings and Relationships, (3) Daily Life, (4) Information and Activities, (5) Education and (6) Work. Notably, the CNQ-YP has been well-validated by published literature, indicating strong psychometric properties [41,42]. All six dimensions of the CNQ-YP in this study reported satisfactory internal consistency, with Cronbach’s alphas ranging from 76% to 82%.

#### 2.3.3. Self-Rated Health (SRH)

SRH was measured by a single question asking the participant, “In general, would you say your health is?” A participant responded to a 5-point Likert scale of “5 = Excellent”, “4 = Very Good”, “3 = Good”, “3 = Fair”, or “1 = Poor” to indicate their perceived health status. Given the distribution of this variable, SRH was regrouped into “Excellent or Very Good”, “Good”, or “Poor or Fair”.

### 2.4. Statistical Analysis Plan

Data analysis was conducted in R statistical software (version 4.2.1). Two research assistants first conducted descriptive statistics for the entire sample, reporting means and standard deviations for continuous variables; frequency and percentage for nominal variables. Then, descriptive statistics were reported separately using participants’ SO/GI combined as the group variable. Finally, we evaluated the between-group difference (SGM versus non-SGM groups) for unmet needs and SRH to determine if there were any significant differences. We conducted the independent sample’s *t*-test for continuous outcomes (using the Levene’s Test for Equality of Variances to determine the *p* value) and the chi-square test for nominal variables. Given the distribution of the SRH variable, we used multinomial logistic regression to evaluate the relationship between unmet cancer care needs and SRH (recoded into: 0 = poor or fair health, 1 = good health, or 2 = very good or excellent health). Important covariates were controlled for, including age, race/ethnicity, SO/GI (SGM versus non-SGM), and cancer treatment stage. We explored the possible mediation role of sexual orientation/gender identity by creating a series of interaction terms between significant factors correlated with SRH.

## 3. Results

### 3.1. Descriptive Statistics of the Study Population

Key findings indicate that SGM participants reported greater mean unmet needs across all dimensions overall, and significantly greater unmet needs in areas of Feelings and Relationships, Information and Activities, and Education. Findings also reveal a statistically significant difference in SRH between SGM versus non-SGM participants, with SGM participants reporting poorer SRH.

Table 1 displays the descriptive statistics of the study population. A total of *n*= 324 eligible AYAs with cancer completed and returned valid surveys. Participants reported an average age of 30.22 years old (SD = 6.50) and ranged from 16 to 39 years old. Most participants (28.4%) were long-term survivors who, at the time of the study, were more than 5 years post-treatment. The second largest treatment group were AYAs who were 1–3 years post-treatment (26.2%), followed by those who were 3–5 years post-treatment (17.3%). The two smaller treatment groups were AYAs within 1 year post-treatment (14.2%) and about 13.9% were in active treatment at the time of the study.

Most participants identified as non-Hispanic White (*n* = 289, 89.2%), with the remaining participants identifying as Black/African American (*n* = 8; 2.5%), Asian (*n* = 8; 2.5%), Native Hawaiian or other Pacific Islander (*n* = 8; 2.5%), American Indian or Alaska Native (*n* = 1; 0.3%), Hispanic/Latino (*n* = 5; 1.6%), Bi/Multi-racial (*n* = 3; 0.9%), or Another race/ethnicity not listed (*n* = 2; 0.6%). SGM participants (i.e., those who selected SO/GI items other than cisgender and/or heterosexual) comprised *n* = 45. Nearly 8.95% (*n* = 29) participants identified as both non-cisgender and non-heterosexual.

For gender, most participants (*n* = 215; 66.4%) identified as Woman/Girl (participants who selected Woman/Girl, or Woman/Girl AND Cisgender). Ninety-four (29%) identified as Man/Boy (participants who selected Man/Boy, or Man/Boy AND cisgender). One (0.3%) identified as Transmasculine, one (0.3%) identified as a Man/Boy AND Transmasculine, and several participants identified as Nonbinary (*n* = 7; 2.2%).

Regarding sexual orientation, most participants identified as Straight/heterosexual (*n* = 279; 86.4%), followed by seventeen (5.3%) who identified as Bisexual, five (1.6%) as Pansexual, six (1.5%) as Gay, three (0.9%) as Queer, and one (0.3%) as Lesbian, and the remainder are outlined in Table 1.

For SRH, over one-third of the study participants reported Good overall health (*n* = 120, 37%) or Very Good overall health (*n* = 106, 32.7%), while sixty-three participants (19.4%) reported Fair overall health, whereas twenty-eight participants (8.6%) reported Excellent overall health and seven participants (2.2%) reported Poor overall health.

### 3.2. Between-Group Differences in Self-Rated Health (SRH) and Unmet Cancer Needs

Table 2 shows the between-group differences in SRH and unmet cancer needs between SGM and non-SGM participants. Overall, the difference in SRH between SGM and non-SGM AYA participants was statistically significant, with $\chi^{2}\left(4\right)$ = 15.95, *p* = 0.031. The result of the chi-square test revealed that SGM AYA cancer survivors reported significantly lower SRH when compared to their counterparts who are non SGM AYAs with cancer.

In addition, in terms of unmet cancer needs, SGM participants reported higher needs across all dimensions as shown by the higher means of all dimensions compared to non-SGM participants. Furthermore, SGM participants reported significantly greater needs than their non-SGM counterparts in areas of Feelings and Relationships, *t*(314) = −2.111, *p* = 0.036, Information and Activities, *t*(314) = −2.594, *p* = 0.009, and Education, *t*(207) = −3.289, *p* < 0.001. Between-group differences were statistically non-significant in areas of Treatment Environment and Care, Daily Life, and Work.

### 3.3. The Relationship between Unmet Cancer Needs and SRH

Table 3 demonstrates the relationship between unmet cancer care needs and SRH. For both SGM and non-SGM AYAs with cancer, an AYA cancer survivor’s daily life needs were significantly associated with their SRH. Specifically, for each unit increase in an AYA cancer survivor’s unmet daily life needs, they are 9.5% less likely to report good health versus fair or poor health, OR = 0.905, 95% CI [0.839, 0.977], *p* < 0.01. For each unit increase in their unmet daily life needs, an AYA is 11.5% less likely to report very good or excellent health versus fair or poor health, OR = 0.885, 95% CI [0.816, 0.961], *p* < 0.001. In addition, the unmet work needs are significantly associated with SRH. Interestingly, for each unit increase in their unmet work needs, an AYA is 1.21 times more likely to report good health versus fair or poor health, OR = 1.210, 95% CI [1.009, 1.451]. Similarly, for each unit increase in their unmet work needs, an AYA is 1.225 times more likely to report excellent or very good health versus fair or poor health, OR = 1.225, 95% CI [1.016, 1.476], *p* < 0.05. Moderator analysis evaluating the moderating role of SGM identities on the relationship between the unmet daily life needs and SRH, and between the unmet work needs and SRH, did not indicate SGM status being a significant moderator.

## 4. Discussion

The current study provides novel insights into the relationship between unmet needs and self-rated health among SGM AYA cancer survivors. Additionally, our study sheds light on the experiences of a population of cancer survivors about whom little is known: SGM AYAs. We hypothesized that SGM AYAs would have significantly greater unmet needs across all six domains and lower SRH compared to their non-SGM counterparts.

Our finding that SGM AYAs have greater mean unmet needs across all domains builds on the literature which shows that SGM populations are more likely to report unmet medical needs [46]. SGM populations as a whole report a lack of knowledge on the part of health care providers about their health care needs [47]. Specifically, in oncology, most provider knowledge is centered on cancer screening and prevention. Further, transgender and gender-diverse patients have additional distinct needs [48]. In terms of health care delivery, distinct needs for transgender and gender-diverse AYAs include specialized counsel for fertility preservation decision making in light of emergent gender-diverse identities [49]; guidance regarding the contra indications of gender-affirming hormone use before, during, or after chemotherapy [17]; and therapeutic counsel about gender dysphoria arising from disruption to gender-affirming hormones if indicated by therapeutic or supportive treatments [50,51,52]. Future research into transgender and gender-diverse AYAs’ unmet health care needs is urgently needed.

The finding that SGM AYAs have significantly greater unmet needs regarding Feelings and Relationships, Information and Activities, and Education aligns with the literature that has shown that SGM cancer survivors across all ages are more likely to experience psychosocial distress than non-SGM cancer survivors [22]. In another study of adult SGM cancer survivors, the majority reported unmet needs regarding feelings and relationships, and indicated a significant need for mental health resources [53]. In terms of supportive relationships, SGM young people frequently face family-of-origin rejection after coming out. As such, SGM AYAs may lack supportive familial networks [14]. Therefore, family-of-origin support for SGM AYAs must not be presumed by the health care team. Targeted screenings and interventions for mental health, financial, and other instrumental resource needs (e.g., health care navigation; housing) are advised for SGM AYAs. Furthermore, family rejection is predictive of self-harm and suicide for SGM AYAs, especially transgender and gender-diverse individuals [54]. Cancer patients are an at-risk group for self-harm and suicidal behaviors [55]. Therefore, SGM AYAs may be a high-risk group for self-harm, and future research is advised to evaluate this potential risk.

The timing of our data collection is notable in terms of our findings that SGM AYAs expressed greater unmet educational needs. Past research has highlighted that the timing of a cancer diagnosis during adolescence and young adulthood disrupts the achievement of typical educational milestones [56,57]. Recent research published from data collected during the COVID-19 pandemic has also shown that AYAs in general are also more likely to experience educational disruptions than older adults [58,59]. This may be partly explained by the social/physical distancing that resulted from the pandemic, and educational institution closures, which have been shown to have disproportionately affected SGM AYAs who endorsed greater distress and isolation, with fewer coping resources than non-SGM AYAs in one study [60]. Currently, there are no interventions among SGM AYAs with cancer to improve educational or employment outcomes [61]. Among AYA cancer survivors in general, and SGM AYAs in particular, educational support is critically needed.

The finding that SGM AYAs have significantly lower SRH compared to non-SGM AYAs is a notable finding and supports past work reporting that SGM adults endorse worse SRH than non-SGM adults [62,63]. Poor SRH has been shown to be related to minority stress components including discrimination, victimization, concealment of SGM status, and structural stigma [64]. In one study, disclosure of SGM status to oncology providers was associated with better self-reported health among SGM adults [65]. Our past work has highlighted that current AYAs live in a society with fluid sexual attractions and gender expressions, where one cannot make assumptions about goals for relationships and children, and when navigating illnesses such as cancer, SGM AYAs often seek refuge in a “chosen family” [66,67]. These intersecting identities brought forth by both illness, AYA and SGM status warrant further research on addressing discrimination in the healthcare setting where there is a need for recognition and support of non-heteronormative supportive care models. That is, cultivating SGM-competent cancer research may be connected to addressing unmet needs among SGM AYAs [68].

Finally, we found that for both SGM and non-SGM AYA’s with cancer there is a significant association between unmet daily life needs and SRH, such that those reporting higher unmet daily life needs were more likely to report poorer health. This is of particular importance as SRH has been extensively shown to be predictive of overall mortality [69,70]. Moreover, as SGM AYAs with cancer are posited to have a greater prevalence of unmet needs than their non-SGM counterparts, this finding could indicate the potential for disparity in survival rates. The findings also suggest a significant association between unmet work needs and SRH such that those AYAs with cancer who reported higher unmet work needs were more likely to report better health. This finding—although seemingly counterintuitive—may indicate that AYAs with unmet work needs are well enough to return to work following completion of active treatment and may be struggling to transition back to the workplace for numerous reasons (e.g., “chemo brain”; disrupted work schedule due to surveillance appointments). By the same logic, those reporting poorer health may, in turn, not be primarily focused on work, may be on hiatus from work (i.e., temporarily receiving Social Security Disability Insurance), and/or may be at a point in their cancer trajectory where determining needs surrounding work proves difficult or impossible [71]. Nevertheless, this association should be explored further. Furthermore, researchers are advised to explore how employers may best empower AYA cancer survivors during their transition back into the workplace. Particularly following the COVID-19 pandemic, there has been a surge in the popularity and prevalence of “remote work,” or working from home [72]. We call upon researchers to explore the impact of remote work on the needs of this population to determine what resources will be necessary for successful, supportive, and sustainable reintegration into the workplace.

The current study was limited by a low response rate. Such a limitation may be explained by the timing of data collection in terms of research fatigue following the onset of the COVID-19 pandemic. People affected by disasters—especially vulnerable and marginalized groups—often receive multiple requests for study participation which may lead to participant fatigue and divestment [73]. Accordingly, the representativeness of the sample may have been mitigated by self-selection bias (i.e., those who participated suffered less research fatigue). Recruitment methods may also explain a low response rate. We used postal mail to reach eligible participants because it was the most consistent and reliable form of contact information made available through the cancer registry. However, AYA is a population that relocates residences frequently. Furthermore, this age cohort is more likely to participate in research activity via text message, email, social media, or other interactive digital methods [74]. Future studies are advised to continue innovating toward age-tailored recruitment and retention strategies. Furthermore, cancer registries are also advised to consistently and regularly collect and update phone number and email contact information so that researchers may identify and engage the population through channels that are meaningful to AYAs.

Secondly, while we included respondents from both active treatment and post-active treatment phases, we did not analyze time since diagnosis as a factor in evaluating unmet needs. Future research is advised to explore changes in unmet needs over time, especially given our group’s previous findings which indicated cancer survivors’ mental health needs (e.g., worry) increased over time following the completion of treatment [75].

Finally, given the cross-sectional nature of the data, we were only able to evaluate association but not causality, and future research should consider a longitudinal design to strengthen the implication for causality.

Overall, the findings of the current study underscore how vital it is to understand the unique needs of SGM populations and to work toward highlighting potential targets for future intervention.

## Figures and Tables

**Table 1:** Descriptive Statistics of Adolescents and Young Adults with Cancer (*n* = 324) *.

Name of Variable	Mean/SD OR Frequency (%)
Dimensions of Unmet Needs	
Treatment Environment and Care	3.99/0.61
Feelings and Relationships	2.13/0.89
Daily Life	2.19/0.94
Information and Activity	2.98/0.91
Education	2.97/1.26
Work	3.14/1.44
Age (Years)	30.22/6.50
Cancer Status	
Active treatment	45 (13.9%)
Within 1 year survivor	46 (14.2%)
1–3 years survivor	85 (26.2%)
3–5 years survivor	56 (17.3%)
>5 years survivor	92 (28.4%)
Race/ethnicity	
Black/African American	8 (2.5%)
Hispanic/Latino	5 (1.5%)
Non-Hispanic White only	289 (89.2%)
American Indian or Alaska Native	1 (0.3%)
Another race/ethnicity not listed	2 (0.6%)
Hispanic/Latino and Bi/Multi-racial or ethnicity	1 (0.3%)
Non-Hispanic White and American Indian or Alaska Native	1 (0.3%)
Bi/Multi-racial or ethnicity	1 (0.3%)
Asian American	8 (2.5%)
Native Hawaiian or Other Pacific Islander	8 (2.5%)
Gender	
Woman/Girl	215 (66.4%)
Man/Boy	94 (29%)
Transmasculine	1 (0.3%)
Nonbinary	7 (2.2%)
Woman/Girl and Nonbinary	1 (0.3%)
Woman/Girl and Cisgender	2 (0.6%)
Man/Boy and Transmasculine	1 (0.3%)
Man/Boy and Cisgender	3 (0.9%)
Sexual Orientation	
Lesbian	1 (0.3%)
Gay	6 (1.5%)
Bisexual	17 (5.2%)
Pansexual	5 (1.5%)
Straight/Heterosexual	279 (86.4%)
Queer	3 (0.9%)
Another sexual orientation not listed	1 (0.3%)
Prefer not to say	5 (1.6%)
Lesbian and Queer	1 (0.3%)
Gay and Queer	1 (0.3%)
Bisexual and Pansexual	2 (0.6%)
Bisexual and Straight/Heterosexual	1 (0.3%)
Bisexual and Queer	1 (0.3%)
Pansexual and Queer	1 (0.3%)
Self-Rated Health	
Poor	7 (2.2%)
Fair	63 (19.4%)
Good	120 (37.0%)
Very good	106 (32.7%)
Excellent	28 (8.6%)

* mean/SD for continuous variables, and *n* (%) for categorical variables.

**Table 2:** Between Group Differences of Self-Rated Health *.

Name of Variable	SGM ** (*n* = 45)	Non-SGM ** (*n* = 279)	Difference
Dimensions of Unmet Needs			
Treatment Environment and Care	4.027/0.640	3.990/0.610	*t*(309) = −0.376, *p* = 0.707
Feelings and Relationships	2.460/0.940	2.144/0.928	*t*(314) = −2.111, *p* = 0.036
Daily Life	2.327/0.892	2.109/0.889	*t*(229) = −1.116, *p* = 0.276
Information and Activities	3.307/0.928	2.931/0.895	*t*(314) = −2.594, *p* = 0.009
Education	3.634/1.086	2.849/1.249	*t*(207) = −3.289, *p* < 0.001
Work	3.250/1.240	3.121/1.466	*t*(278) = −0.527, *p* = 0.599
Age (years)	28.568/6.460	30.491/6.479	*t*(317) = 1.846, *p* = 0.066
Cancer Status			--
Active treatment	6 (13.3%)	39 (14%)	
Within 1 year survivor	6 (13.3%)	40 (14.3%)	
1–3 years survivor	13 (28.9%)	72 (25.8%)	
3–5 years survivor	9 (20.0%)	47 (16.8%)	
>5 years survivor	11 (24.4%)	81 (29%)	
Race/ethnicity			
Black/African American	2 (4.4%)	6 (2.2%)	--
Hispanic/Latino	0 (0.0%)	5 (1.8%)	
Non-Hispanic White only	40 (88.9%)	249 (89.2%)	
American Indian or Alaska Native	0 (0.0%)	1 (0.4%)	
Asian American	1 (2.2%)	7 (2.5%)	
Native Hawaiian or Other Pacific Islander	2 (4.4%)	6 (2.2%)	
Another race/ethnicity not listed	0 (0.0%)	2 (0.7%)	
Hispanic/Latino and Bi/Multi-racial or ethnicity	0 (0.0%)	1 (0.4%)	
Non-Hispanic White and American Indian or Alaska Native	0 (0.0%)	1 (0.4%)	
Non-Hispanic Asian or Bi/Multi-racial or ethnicity	0 (0.0%)	1 (0.4%)	
Self-Rated Health			
Poor	4 (8.9%)	3 (1.1%)	$\chi^{2}\left(4\right)$ = 15.95, *p* = 0.031
Fair	12 (26.7%)	51 (18.6%)	
Good	17 (37.8%)	103 (36.9%)	
Very good	11 (24.4%)	95 (33.9%)	
Excellent	1 (2.2%)	27 (9.5%)	

* mean/SD for continuous variables, and *n* (%) for categorical variables. ** SGM = Sexual and gender minority.

**Table 3:** Multinomial Logistic Regression ^†,1^.

	Reference Group: Fair or Poor Health			
	Good Health		Excellent or Very Good Health	
	OR	95% CI	OR	95% CI
Non-SGM (versus SGM)	0.709	[0.097, 5.175]	4.056	[0.347, 47.415]
Unmet cancer care needs				
Treatment environment and care	0.989	[0.955, 1.025]	1.008	[0.971, 1.045]
Daily life	0.905 **	[0.839, 0.977]	0.885 **	[0.816, 0.961]
Feelings and relationships	1.025	[0.957, 1.097]	0.958	[0.886, 1.036]
Information and activities	0.938	[0.794, 1.109]	0.937	[0.785, 1.118]
Education	1.033	[0.870, 1.226]	1.058	[0.885, 1.265]
Work	1.210 *	[1.009, 1.451]	1.225 *	[1.016, 1.476]

^1^ Other variables controlled in the model included age, cancer treatment stage, and race/ethnicity. We also evaluated the potential moderating role of SGM versus non-SGM for significant predictors in the model, none were statistically significant, thus not presented in the model. ^†^ *p* < 0.06, * *p* < 0.05, ** *p* < 0.01.

## Data Availability

Raw data can be made available upon reasonable request.
